# Safety and preliminary efficacy of Disitamab Vedotin combined with Toripalimab and concurrent radiotherapy in bladder-preserving treatment for HER2-positive invasive bladder cancer

**DOI:** 10.3389/fonc.2026.1880457

**Published:** 2026-07-01

**Authors:** Han Chen, Nan Liu, Xinyu Chen, Peng Xian, Jun Li, Junyong Dai, Gangjun Yuan, Fang Yuan

**Affiliations:** Chongqing University Cancer Hospital, Chongqing, China

**Keywords:** antibody-drug conjugate, bladder preservation, concurrent radiotherapy, immune checkpoint inhibitor, invasive bladder cancer

## Abstract

**Aims:**

To explore the safety and preliminary efficacy of Disitamab Vedotin (DV) combined with Toripalimab (T) and concurrent radiotherapy regimen (DVTR) in the comprehensive bladder-preserving treatment for HER2-positive invasive bladder cancer patients.

**Material:**

A total of 10 invasive bladder cancer patients who met the indication for radical cystectomy (RC) but refused the surgery were enrolled from January 2023 to Sep 2025, with follow-up extended until March 2026. All patients underwent maximum transurethral resection of bladder tumor (mTURBT)+/-neoadjuvant therapy with DV plus T after postoperative pathology confirmed HER2 positivity. Concurrent radiotherapy was administered combined with synchronous DV plus T treatment. Patients were followed up until March 2026 or death after the end of treatment.

**Results:**

The overall treatment completion rate was 90% (9/10), with 1 patient only receiving 46 Gy of radiotherapy due to personal reasons. The incidence of grade ≥3 adverse reactions during treatment was 10% (1/10), which was leukopenia. The common grade 1–2 adverse reactions were radiation cystitis (9/10) and hand-foot numbness (2/10); hemorrhagic cystitis caused by radiation occurred in 2 patients (2/10) at the 20th and 22nd months after treatment, respectively. At the last follow-up, the median follow-up time was 17.2 months (range, 6–31 months). The bladder preservation success rate was 90% (9/10); the patient who did not complete radiotherapy had T3 recurrence at 5 months, underwent salvage RC and recovered normally, however, died of intestinal obstruction complications 13 months later. The event-free survival rate was 80% (8/10); 1 patient had T1 recurrence at 7 months after treatment and continued bladder-preserving treatment for 31 months.

**Conclusion:**

As a novel strategy for comprehensive bladder-preserving treatment, the DVTR regimen based on DV combined with T and concurrent radiotherapy has initially shown acceptable safety and preliminaryly reliable efficacy in this study. It provides a potential bladder-preserving option for patients with HER2-positive invasive bladder cancer regardless of PD-L1 status, though this conclusion requires validation in larger cohorts,which is worthy of further investigation with an expanded sample size.

## 1 Introduction

Radical cystectomy (RC) combined with pelvic lymph node dissection is the standard treatment for muscle-invasive bladder cancer (MIBC) and very high-risk non-muscle-invasive bladder cancer (VHR-NMIBC). However, the surgery is highly invasive, and the significant changes in quality of life caused by postoperative urinary diversion lead some patients to strongly refuse radical surgery (1). Trimodal therapy (TMT), consisting of maximum transurethral resection of bladder tumor (mTURBT) combined with concurrent chemoradiotherapy, is currently the bladder-preserving regimen with the most evidence-based medical support (2). Traditional TMT usually adopts radiotherapy combined with low-dose cisplatin, but its application is limited in patients with renal insufficiency, hearing impairment or intolerance to cisplatin toxicity, with gemcitabine often used as a cisplatin alternative (2, 3).

In recent years, antibody-drug conjugates (ADCs) and immune checkpoint inhibitors (ICIs) have achieved breakthrough efficacy in the treatment of advanced urothelial carcinoma (4, 5). Disitamab Vedotin (DV) is an ADC targeting HER2, and Toripalimab (T) is a PD-1 inhibitor, both of which have obtained indications for the second-line treatment of advanced urothelial carcinoma based on pivotal clinical trials (4, 5). DV requires high HER2 expression, while T does not require PD-L1 expression testing. A recent phase III randomized controlled trial showed that the combination of the two agents significantly improved the anti-tumor activity compared with platinum-based chemotherapy as first-line treatment for advanced urothelial carcinoma (6). Theoretically, the combination of ADC and ICI can not only directly kill tumor cells and induce immunogenic cell death but also reverse the tumor immune microenvironment. Its combination with the local killing effect of radiotherapy and the abscopal effect induced by immune activation may provide a new therapeutic combination for bladder preservation. This study aimed to observe the safety and preliminary efficacy of the DVTR regimen (DV combined with T and concurrent radiotherapy) in bladder-preserving treatment and initially explore the predictive biomarkers for efficacy.

## 2 Materials and methods

### 2.1 Study subjects

Patients with bladder cancer admitted to The Affiliated Tumor Hospital of Chongqing University from January 2023 to April 2025 were enrolled.

Inclusion criteria: ① Pathologically confirmed invasive urothelial carcinoma of the bladder, which may be accompanied by adverse pathological subtypes and carcinoma *in situ*; ② Clinical stage cT1-4aN0M0 (8th edition of AJCC), including VHR-NMIBC and MIBC; ③ Patients strongly refused RC or were evaluated as suitable for bladder-preserving treatment by multidisciplinary team (MDT); ④ No gender restriction, aged 18–85 years; ⑤ Cardiac, hepatic, renal and bone marrow hematopoietic functions tolerable to treatment; ⑥ Positive HER2 expression by immunohistochemistry (IHC), regardless of PD-L1 expression.

Exclusion criteria: ① Distant metastasis or regional lymph node metastasis; ② A history of severe hypersensitivity to monoclonal antibodies; ③ Active autoimmune diseases; ④ Definite contraindications to radiotherapy.

**Figure 1:**
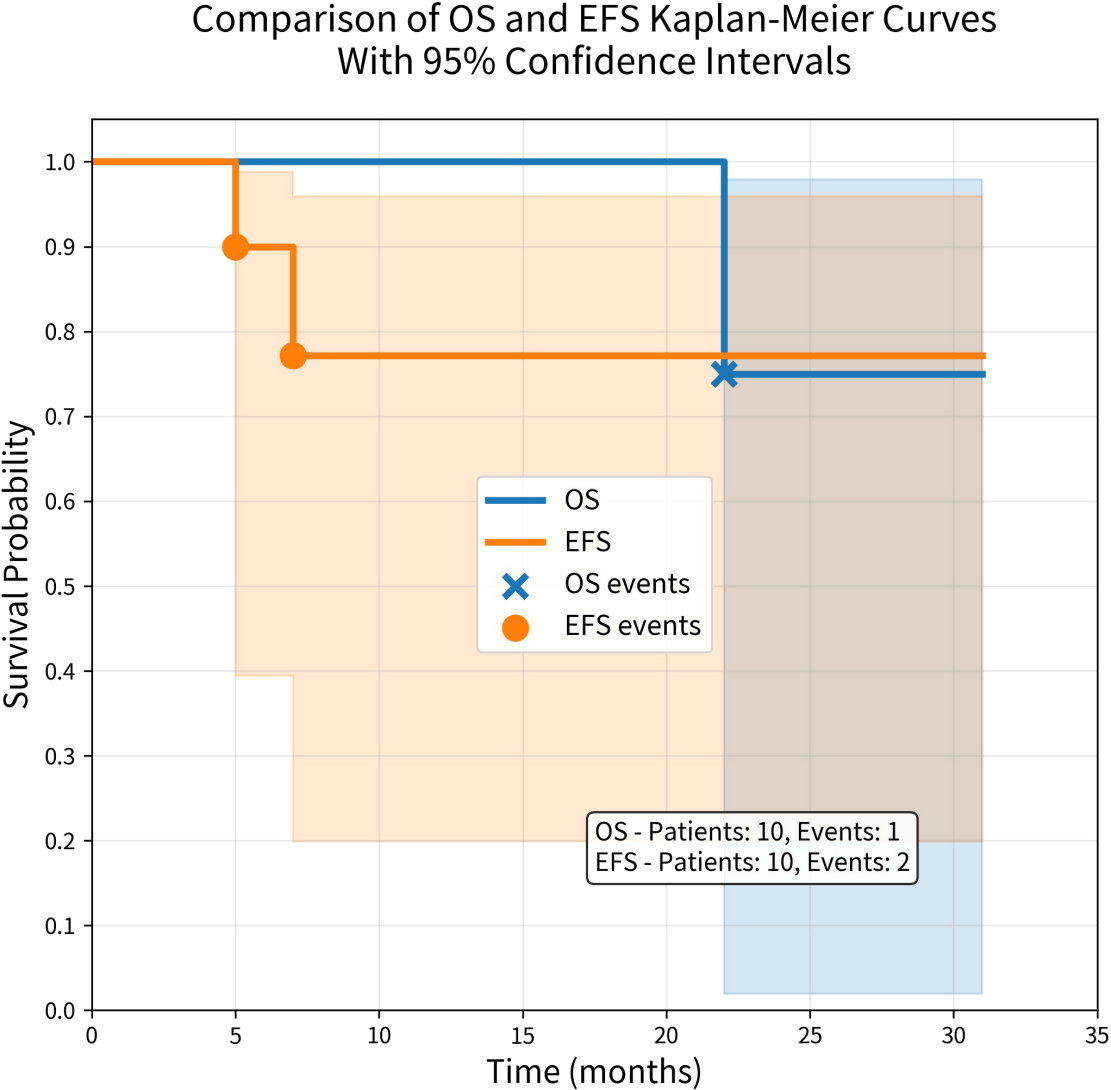
Study flow chart of DVTR regimen for bladder-preserving treatment. This flow chart depicts the entire treatment and follow-up protocol of Disitamab Vedotin combined with Toripalimab and concurrent radiotherapy (DVTR) for HER2-positive invasive bladder cancer patients undergoing bladder-preserving treatment. All patients first received maximum transurethral resection of bladder tumor (mTURBT), followed by optional neoadjuvant therapy with Disitamab Vedotin (2.0 mg/kg, IV, D1) plus Toripalimab (240 mg, IV, D1) every 2 weeks for 2–6 cycles (median 3 cycles) with multiparametric magnetic resonance imaging (mpMRI) before and after treatment. Concurrent intensity-modulated radiotherapy (IMRT) was initiated after secondary TURBT/cystoscopy confirmed no residual tumor, with the irradiation range covering the whole bladder and regional lymph drainage area at a total dose of 60–64 Gy (2 Gy/fraction) or 55 Gy (2.75 Gy/fraction ×20). Disitamab Vedotin plus Toripalimab was synchronously administered every 2 weeks during radiotherapy, with weekly adverse reaction assessment. The first efficacy evaluation was conducted at 3 months post-treatment (cystoscopy, urine cytology, chest-abdomen-pelvis CT/mpMRI), and regular reexaminations were performed every 3 months thereafter. Tumor recurrence was defined as pathologically confirmed new lesions, and events included local bladder recurrence, distant metastasis, death, or salvage radical cystectomy (RC).

This study was approved by the Hospital Ethics Committee (JG-ZN-001-01, 2017-042), and all patients signed informed consent forms. (**Figure 1**).

### 2.2 Treatment and follow-up regimen

**Figure 1** illustrates the overall study design and treatment flow.

#### 2.2.1 Initial treatment and evaluation

All patients first underwent maximum TURBT in accordance with current clinical guidelines for bladder cancer (7). According to HER2 expression status after surgery, patients were recommended to receive neoadjuvant therapy with DV (2.0 mg/kg, intravenous infusion, Day 1) combined with T (240 mg, intravenous infusion, Day 1), once every 2 weeks, with a median of 3 cycles (range, 2–6 cycles). Multiparametric magnetic resonance imaging (mpMRI) of the bladder was performed before and after treatment.

#### 2.2.2 Concurrent radiotherapy

Regardless of neoadjuvant therapy, concurrent radiotherapy was initiated after secondary TURBT or cystoscopy confirmed no definite residual tumor. Intensity-modulated radiotherapy (IMRT) was adopted, with the irradiation range including the entire bladder and regional lymph drainage area. The planned total dose was 60–64 Gy (2.0 Gy per fraction) or an equivalent biological dose fractionation regimen (55 Gy, 2.75 Gy per fraction for 20 fractions). Synchronous systemic DV plus T treatment (at the same dose as above) was administered once every 2 weeks during radiotherapy. Adverse reactions were evaluated weekly during treatment.

#### 2.2.3 Efficacy evaluation and follow-up

The first efficacy assessment was conducted at 3 months after the end of treatment, including cystoscopy, urine cytology, and chest-abdomen-pelvis computed tomography (CT)/mpMRI. Reexaminations were performed every 3 months thereafter. Tumor recurrence was defined as newly detected lesions by cystoscopy or imaging confirmed by pathology. Events were defined as local bladder recurrence, distant metastasis, death, or salvage RC.

### 2.3 Observation indicators

#### 2.3.1 Safety

All adverse events during treatment were recorded and graded according to the *Common Terminology Criteria for Adverse Events (CTCAE)* v5.0.

#### 2.3.2 Efficacy

The primary efficacy indicators included bladder preservation rate (proportion of patients without salvage RC), event-free survival (EFS) rate, and overall survival (OS) rate.

#### 2.3.3 Pathological and biomarker analysis

Pre-treatment tumor tissue samples of patients were collected for IHC detection of HER2, PD-L1, p53, Ki-67 and other biomarkers.

PD-L1 immunohistochemical staining in tumor tissue was performed using the VENTANA PD-L1 (SP263) immunohistochemistry method. The PD-L1 (SP263) protein detection kit and the fully automated immunohistochemistry staining instrument (BenchMark ULTRA) were both purchased from Roche. PD-L1 expression levels were determined by the proportion of positive tumor cells and immune cells in the invasive carcinoma tissue. PD-L1 high expression was defined as tumor cell membrane positivity rate (TC) ≥ 25%; or immune cell positivity rate (IC) ≥ 25% when ICP (Total area of tumor-associated immune cells/Total tumor area) > 1%; or IC positivity rate = 100% when ICP = 1% (6).HER2 expression was assessed by IHC according to the following criteria: 0 (no staining), 1+ (faint/barely perceptible staining in ≥10% tumor cells), 2+ (weak-to-moderate complete membrane staining in ≥10% tumor cells), and 3+ (strong complete membrane staining in ≥10% tumor cells).

### 2.4 Statistical methods

SPSS 26.0 software was used for data analysis. Measurement data were expressed as mean ± standard deviation or median (range). Survival analysis was performed using the Kaplan-Meier method. Given the small sample size *n=10* and limited number of events *n=2*, survival estimates are exploratory and associated with wide 95% confidence intervals. As this was a preliminary exploratory study with a small sample size, descriptive statistical analysis was the main method adopted.

## 3 Results

### 3.1 Baseline characteristics and treatment status of patients

A total of 10 patients were enrolled in this study, and their baseline characteristics and treatment status are shown in **Table 1**. The median age was 70.1 years (range, 61–81 years), including 9 males (90%) and 1 female (10%). There were 6 newly diagnosed patients (2 with cT3, 4 with cT1) and 4 recurrent patients (1 with cT4a, 1 with cT2, 1 with cT1, and 1 with cT1 after bacillus Calmette-Guérin (BCG) failure). The pre-treatment VI-RADS score of bladder mpMRI was 5 points in 5 cases, 4 points in 3 cases, and 1 point in 2 cases. After neoadjuvant immunotherapy combined with ADC treatment, the mpMRI scores of all 8 patients decreased to 0 points.Neoadjuvant cycles ranged from 2 to 6; all 8 patients achieved complete radiological response (mpMRI score 0) regardless of cycle number, suggesting no clear correlation between cycle count and response in this small cohort.

**Table 1:** Baseline characteristics, treatment regimens and completion status of patients (n=10).

Variable	Value/Case number (%) or Median (range)
Demographic characteristics	
Median age (years)	70.1 (61-81)
Gender (Male/Female)	9 (90%)/1 (10%)
Clinicopathological characteristics	
Clinical stage (cT1/cT2/cT3/cT4a)N0M0	6 (60%)/1 (10%)/2 (20%)/1 (10%)
Primary/Recurrent	6 (60%)/4 (40%)
Pre-treatment VI-RADS score (5/4/1)	5 (50%)/3 (30%)/2 (20%)
Treatment regimen	
Received neoadjuvant therapy (NAT)	8 (80%)
mpMRI score decreased to 0 after NAT	8/8 (100%)
Radiation dose (60 Gy/Other*)	7 (70%)/3 (30%)
Treatment completion status	
Overall treatment completion rate	9/10 (90%)

*Other radiation doses included 55 Gy (1 case), 64 Gy (1 case), and 46 Gy (1 case, incomplete treatment).

### 3.2 Treatment-related adverse reactions

The adverse reactions observed during treatment are shown in **Table 2**, with overall controllable safety. The overall treatment completion rate was 90% (9/10), and 1 patient only completed 46 Gy of radiotherapy due to personal reasons. The incidence of treatment-related grade ≥3 adverse events was 10% (1/10), which was grade 3 leukopenia and recovered after symptomatic treatment with recombinant human granulocyte colony-stimulating factor. Grade 1–2 adverse events were common, including radiation cystitis in 9 cases (90%) manifested as frequent micturition and urgency, leukopenia in 8 cases (80%), hand-foot numbness in 2 cases (20%) considered to be related to DV, and transient radiation proctitis in 3 cases. During follow-up, 2 patients developed delayed hemorrhagic cystitis caused by radiation at the 20th and 22nd months after treatment, respectively, and recovered after pathological confirmation and hemostatic treatment by TURBT. No treatment-related death occurred. The incidence of grade 3+ leukopenia (10%) and radiation cystitis (90%) was consistent with prior reports of radiotherapy alone or combined with single-agent ICIs/ADCs; hand-foot numbness (20%) was comparable to rates reported with single-agent disitamab vedotin. No unexpected additive toxicity was observed in this combination regimen.

**Table 2:** Incidence of treatment-related adverse reactions (n=10).

Type of adverse reaction	Grade 1–2 [Case (%)]	Grade 3–4 [Case (%)]
Hematological toxicity		
Leukopenia	8 (80%)	1 (10%)
Non-hematological toxicity		
Radiation cystitis	9 (90%)	0
Hand-foot numbness	2 (20%)	0
Radiation proctitis	3 (30%)	0
Severe adverse events		
Delayed hemorrhagic cystitis	2 (20%) (20–22 months after treatment)	0
Treatment-related death	0	0

### 3.3 Treatment outcomes and survival

**Figure 2:**
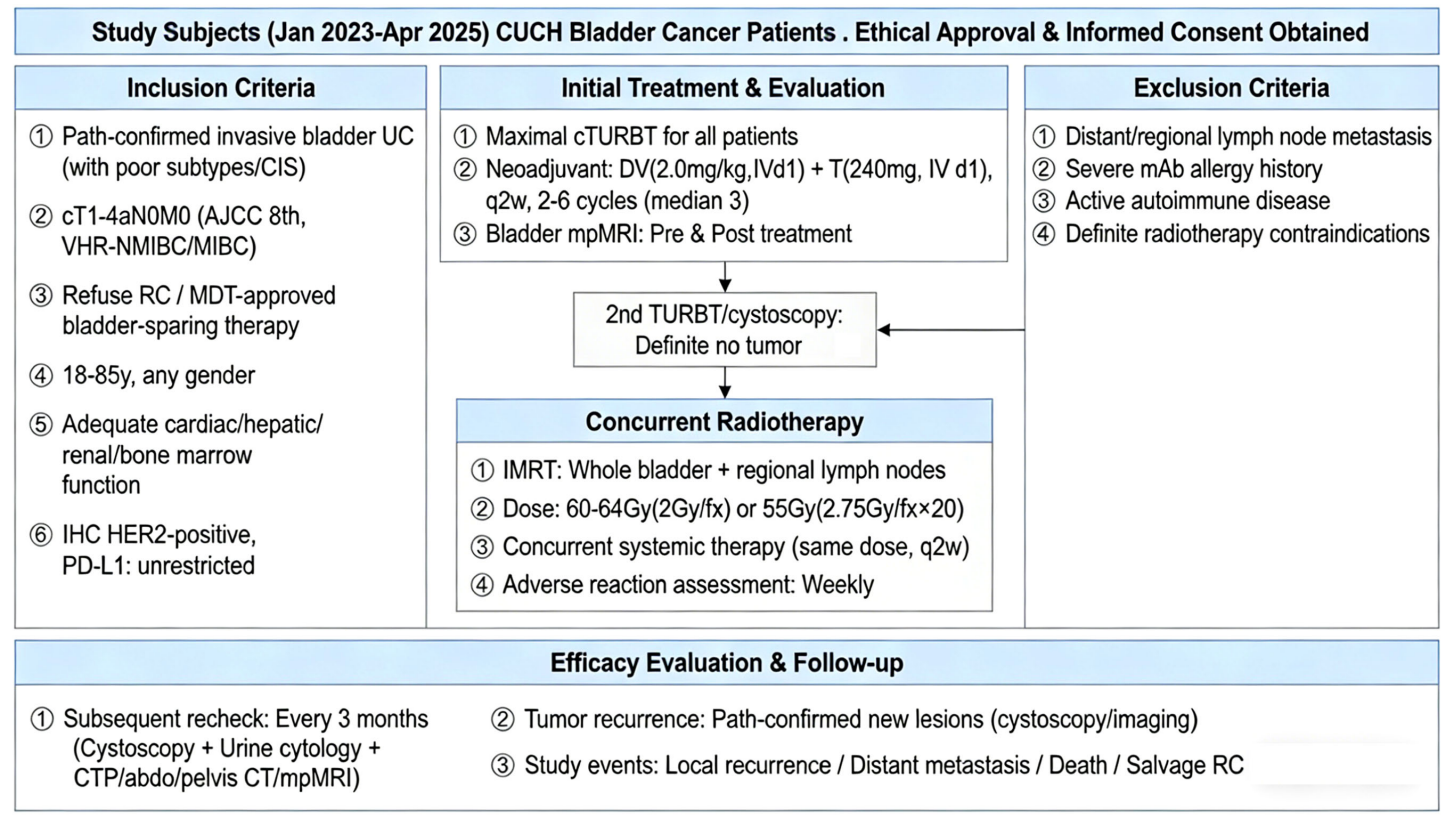
Swimmer plot of follow-up outcomes in patients treated with DVTR regimen. This swimmer plot shows the follow-up duration (months) and clinical events of 10 HER2-positive invasive bladder cancer patients after DVTR regimen treatment, with a follow-up cutoff date of March 2026 (follow-up range: 6–31 months). The horizontal bar length represents the actual follow-up time for each patient (P1–P10). Blue bars indicate patients who completed the full DVTR treatment course, and red bars indicate the patient who did not complete radiotherapy (only 46 Gy). Different symbols mark the occurrence time of clinical events: ★ = tumor recurrence, △ = radiation cystitis hemorrhage, □ =Other Severe Event( radiation cystitis, leukopenia, hand-foot numbness ),◆ = death, ● = grade ≥3 treatment-related adverse events. No symbol indicates no relevant adverse events or clinical events during the follow-up period. Key outcomes: 90% bladder preservation rate and 80% event-free survival rate were achieved in this cohort.

**Figure 3:**
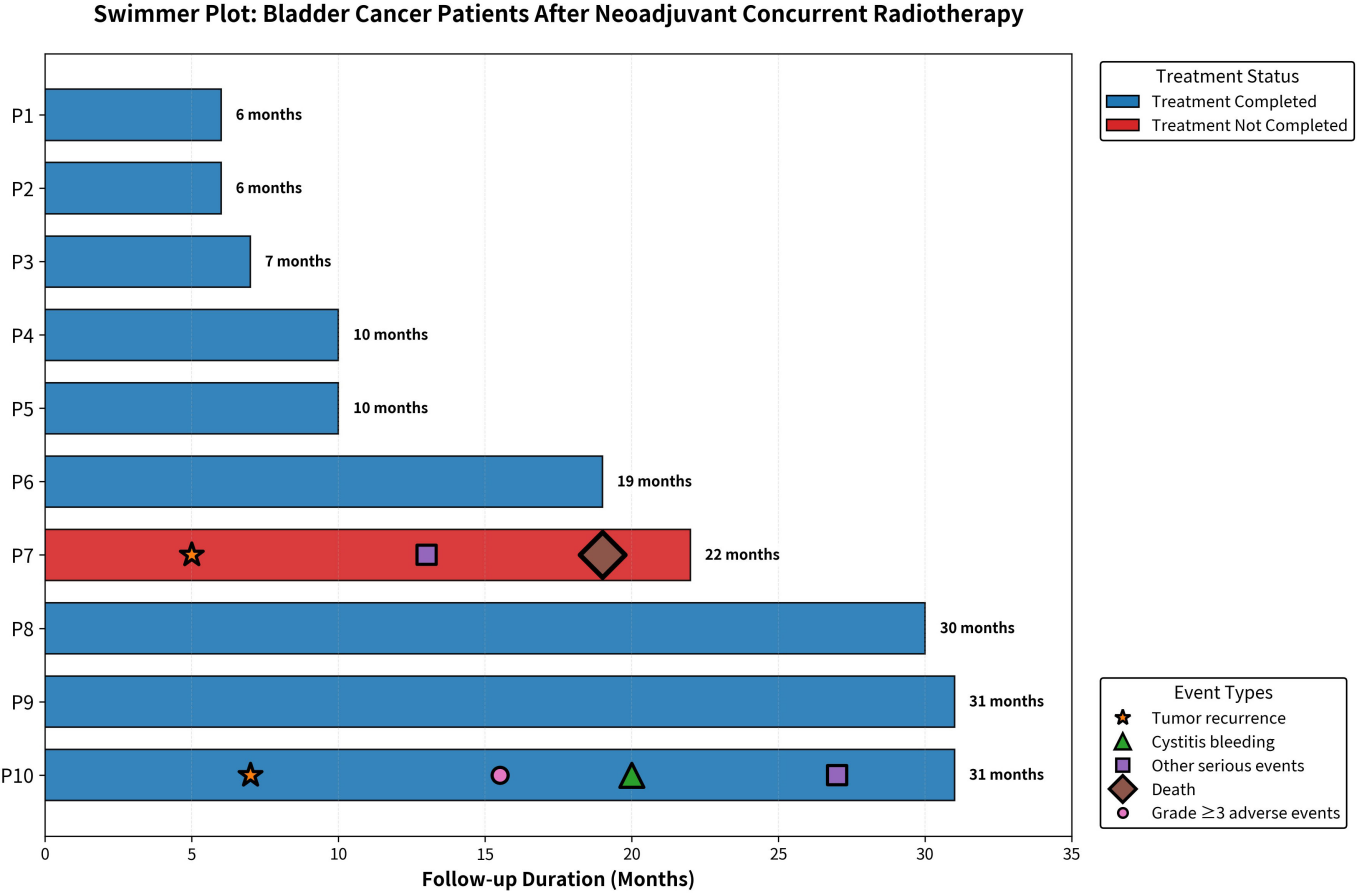
Comparison of overall survival (OS) and event-free survival (EFS). Direct comparison of OS (overall survival) and EFS (event free survival) Kaplan-Meier curves in 10 patients with DVTR. Solid blue line = OS, solid orange line = EFS, with respective 95% CIs (light shading). and the shaded band indicates the 95% confidence interval calculated using the log-log transform method. A single death is marked with a blue “X” at 22 months. An event is defined as disease recurrence, which is marked with solid orange circle. The curve shows a decline at 5 and 7 months corresponding to two recurrence events, the median OS and EFS was not reached. The estimated 1-year, 2-year, and 3-year OS rates (with 95% CI) were 100.0% (100.0,100.0), 75.0% (97.9,1.9), and 75.0% (97.9,1.9), respectively. Event-free survival, with the estimated 1-year, 2-year, and 3-year EFS rates (with 95% CI) were 77.1% (95.9,19.8), 77.1% (95.9,19.8), and 77.1% (95.9,19.8), respectively.

At the last follow-up (March 2026) or death, the median follow-up time was 17.2 months (range, 6–31 months) for overall survival, with 5 patients (50%) followed up for more than 1.5 years and 3 for more than 2 years. The treatment outcomes and survival status are shown in **Table 3** and **Figure 2**. The bladder preservation success rate was 90% (9/10), and the total EFS rate was 80% (8/10). The specific events were as follows: 1 patient with cT1 had T1 recurrence at 7 months after treatment, refused salvage RC, received intravesical instillation after TURBT, and had no recurrence or metastasis at 31 months of follow-up; 1 patient with cT2 (incomplete radiotherapy) had T3 recurrence at 5 months after treatment, immediately received salvage RC, and died of sudden and progressive intestinal obstruction 13 months after surgery. This death was considered unrelated to tumor progression but secondary to post-surgical complications. The remaining 8 patients had no signs of recurrence or metastasis. The estimated 1-year, 2-year, and 3-year OS rates (with 95% CI) were 100.0% (100.0,100.0), 75.0% (97.9,1.9), and 75.0% (97.9,1.9), respectively. Event-free survival, with the estimated 1-year, 2-year, and 3-year EFS rates (with 95% CI) were 77.1% (95.9,19.8), 77.1% (95.9,19.8), and 77.1% (95.9,19.8), respectively(Shown in **Figure 3**).

**Table 3:** Treatment outcomes and survival analysis.

Efficacy indicator	Value [Case (%)] or Median (range)
Median follow-up (months)	17.2 (6-31)
Follow-up >2 years	3/10 (30%)
Follow-up >1.5 years	5/10 (50%)
Bladder preservation success rate	9/10 (90%)
Distant metastasis	0/10 (0%)
Event-free survival (EFS) rate	8/10 (80%)
recurrence (T1 stage)	1/10 (10%)
recurrence (T3 stage)	1/10 (10%)*
Overall survival (OS) rate	9/10 (90%)
Disease-related death	1/10 (10%)*

*The patient did not complete concurrent radiotherapy. He had T3 recurrence and underwent salvage RC, and died of intestinal obstruction at 13 months later.

### 3.4 Pathological and biomarker characteristics

Molecular pathological detection of tumor tissues showed that HER2 expression was 2+ (expressed in both invasive and non-invasive components) in 9 patients (90%) and 1+ in 1 patient (10%). Notably, the single HER2 1+ patient achieved complete response, indicating potential activity of DV even in HER2-low disease, which warrants further investigation. The expression pattern of p53 protein is as follows: 4 patients (40%) had strong positive expression (suggesting mutant type) and 6 patients (60%) had weak to moderate positive expression (suggesting wild type), of whom 1 patient who later had recurrence and death had very weak positive expression. Interestingly, all 10 patients had low/negative PD-L1 expression, suggesting that PD-L1 expression status is not associated with the efficacy in DVTR regimen. Given the uniform PD-L1 negativity in this cohort, whether PD-L1 positivity would confer additional benefit remains unknown and requires further study. In addition, three patients were complicated with urothelial carcinoma *in situ*, and 1 with micropapillary subtype, all of whom achieved definite efficacy with DVTR treatment. Detailed information of each patient is shown in **Table 4**.

**Table 4:** Detailed patient characteristics, treatment, and pathological biomarkers (n=10).

Patient No.	Age (y)/Sex	cT Stage	Primary/Recurrent	Neoadjuvant cycles	RT Dose/Concurrent cycles	Treatment completion	HER2 (IHC)	p53 Pattern (Mutant/Wild-type)	Ki-67 (Hotspot, %)	PD-L1 expression	Concurrent CIS	Special notes
1	75/M	T1	Primary	3	60 Gy/4	Yes	2+	60% strong positive (Mutant)	30%	Low	No	T1 recurrence at 7 mo; HCC at 31 mo.
2	61/M	T1	Primary	4	55 Gy/2 (2.75Gy)	Yes	2+	60% weak positive (Wild-type)	40%	Low	Yes	CIS found at second TURBT.
3	81/F	T1	Primary	4	60 Gy/4	Yes	2+	40% weak positive (Wild-type)	80%	Low	Yes	-
4	69/M	T1	Primary	2	60 Gy/4	Yes	2+	80% strong positive (Mutant)	60%	Low	No	Minimal micropapillary component.
5	64/M	T1	Recurrent	0 (None)	60 Gy/4	Yes	1+	30% weak positive (Wild-type)	30%	Low	No	-
6	71/M	T1	Recurrent (BCG failure)	0 (None)	60 Gy/4	Yes	2+	80% strong positive (Mutant)	30%	Low	Yes	-
7	75/M	T3	Primary	4	60 Gy/4	Yes	2+	60% strong positive (Mutant)	70%	Low	No	TMB-H, MSS status.
8	69/M	T4a	Recurrent	3	64 Gy/3	Yes	2+	30% weak-moderate positive (Wild-type)	20%	Low	No	-
9	71/M	T2	Recurrent	4	46 Gy/4	No	2+	5% very weak positive (Wild-type)	40%	Low	No	T3 recurrence at 5 mo; died post-RC 13th month.
10	65/M	T3	Primary	6	60 Gy/4	Yes	2+	40% weak-moderate positive (Wild-type)	70%	Low	No	-

Bladder Preservation Success Rate: 9/10 (90%). The failure case (Patient 9) did not complete radiotherapy (46 Gy), experienced T3 recurrence at 5 months, underwent salvage radical cystectomy (RC), and died 13 months later. Event-Free Survival (EFS) Rate: 8/10 (80%). Events included the recurrence/death in Patient 9 and a T1 recurrence in Patient 1 (managed with intravesical therapy, bladder preserved at 31 months). Major Adverse Events: One patient (Patient 1) experienced Grade 3 myelosuppression. Common Grade 1–2 events were radiation cystitis (9/10) and hand-foot numbness (2/10). Two patients developed delayed hemorrhagic cystitis >20 months post-treatment.

## 4 Discussion

This study is the first to report the safety and preliminary efficacy of the ADC Disitamab Vedotin combined with the PD-1 inhibitor Toripalimab and concurrent radiotherapy in bladder-preserving treatment for bladder cancer. This is a retrospective, single-arm, single-center pilot study with inherent selection bias and limited generalizability. In this elderly patient population who refused RC, the regimen showed a high treatment completion rate (90%) and bladder preservation rate (90%), with positive disease control effects observed in short and medium-term follow-up.

The efficacy of traditional TMT regimens is closely related to tumor sensitivity to cisplatin. DV delivers cytotoxic drugs to HER2-expressing tumor cells through targeted binding, with a different mechanism from traditional chemotherapy. This study highly selected HER2-positive patients, 90% of whom had high HER2 expression (2+), providing a favorable guarantee for the efficacy of DV. The combination with Toripalimab further activates the immune system and produces a synergistic effect with radiotherapy. Notably, although all patients in this study had negative PD-L1 expression, immunogenic cell death induced by ADC may overcome primary immune resistance, and the synergistic effect of radiotherapy and ICI also achieves effective bladder preservation. This is similar to the effect of Toripalimab without PD-L1 expression testing in the second-line treatment of advanced urothelial carcinoma, but the specific mechanism deserves further study (8). The only patient who underwent salvage RC in this study had very weak p53 expression and incomplete radiotherapy, with rapid recurrence and pathological stage progression, suggesting a close correlation between treatment completion and efficacy. Meanwhile, positive p53 expression may indicate the mutant type, which is a positive predictive indicator of the therapeutic response to this radiotherapy-based bladder-preserving regimen, but further research with an expanded sample size and mechanism exploration are needed for verification.

In terms of safety, no unexpected severe adverse reactions occurred during DVTR treatment. The main adverse reactions were radiation cystitis and DV-related hand-foot numbness, all of which were controllable grade 1–2 events. Although 2 cases of delayed hemorrhagic cystitis were observed, the incidence and occurrence time were comparable to those of traditional radiotherapy (9). It is worth noting that this regimen avoids the adverse effects of cisplatin-related nephrotoxicity, ototoxicity and vascular phlebitis, and is particularly suitable for elderly patients or those with complicated renal insufficiency. An 81-year-old elderly patient in this study successfully completed the entire treatment without developing grade ≥2 adverse reactions.

Compared with the traditional TMT regimen (cisplatin/gemcitabine + radiotherapy) (2), this exploratory study showed comparable bladder preservation rate (86.7% vs 90%) and short-term OS rate. This comparison is limited by differences in baseline stage distribution (more VHR-NMIBC in our cohort) and shorter follow-up duration. However, there were differences in patient selection, clinical stage composition and follow-up time between the two studies. The traditional TMT regimen included more patients with cT2 stage (63.3%), while this study was dominated by VHR-NMIBC (60%), and equivalent safety and efficacy were also observed in patients with more advanced cT3-4a stage. The median follow-up time in the traditional TMT regimen was longer (19.5 months), and its 5-year survival data provided more long-term efficacy evidence. The follow-up time of this study is short, and the long-term efficacy and recurrence pattern need to be further observed. Notably, 3 patients with carcinoma *in situ* and 1 with micropapillary subtype (adverse pathological subtypes) were enrolled in this study, all of whom achieved good efficacy, indicating the clinical applicability of this regimen.

This study has several limitations, including a small sample size, short follow-up time, and single-arm exploratory design without direct control. The current results are only for safety confirmation and preliminary research, and definite conclusions need to be confirmed by larger-scale prospective studies. In addition, the optimal radiation dose fractionation mode, the optimal number of combination cycles of Disitamab Vedotin and Toripalimab, the necessity of neoadjuvant therapy, and the biomarker system for predicting efficacy are the key directions to be further explored in the future.

## 5 Conclusion

As a novel strategy for bladder-preserving treatment of HER2-positive very high-risk invasive bladder cancer, the DVTR regimen has shown manageable safety and encouraging preliminary efficacy and is worthy of further controlled clinical studies with an expanded sample size and exploration of a more optimal application mode.

## Data Availability

The original contributions presented in the study are included in the article/supplementary material. Further inquiries can be directed to the corresponding authors.
